# Psoriasis in Obese Adolescents with Diabetes—From Common Molecular Background to Vicious Circle of Metabolic Syndrome—Case Report and Review of Literature

**DOI:** 10.3390/cells14080610

**Published:** 2025-04-17

**Authors:** Angelika Bielach-Bazyluk, Filip Bossowski, Magdalena Skorupska, Hanna Mysliwiec, Artur Tadeusz Bossowski, Iwona Flisiak

**Affiliations:** 1 Department of Dermatology and Venereology, Medical University of Bialystok, 15-540 Bialystok, Poland; hanna.mysliwiec@gmail.com (H.M.);; 2 Students’ Scientific Society at the Department of Dermatology and Venereology, Medical University of Bialystok, 15-540 Bialystok, Poland; 3 Department of Pediatrics, Endocrinology, Diabetology with Cardiology Divisions, Medical University of Bialystok, 15-274 Bialystok, Poland

**Keywords:** psoriasis, type 1 diabetes, obesity, metabolic syndrome, IL-17, double diabetes, insulin resistance

## Abstract

Psoriasis and type 1 diabetes mellitus (T1DM) are chronic autoimmune diseases sharing common immunological pathways, particularly the involvement of interleukin 17 (IL-17), driving Th17-mediated inflammation. This review explores the overlap between psoriasis, obesity, T1DM, and necrobiosis lipoidica (NL), a skin condition associated with diabetes. Obesity exacerbates inflammation through immune cell activation in adipose tissue and the release of proinflammatory adipokines, such as leptin, resistin, and IL-18, which enhance autoimmune responses and insulin resistance. Leptin promotes the differentiation of Th1 and Th17 cells, which are central to autoimmune responses in both psoriasis and T1DM. The coexistence of psoriasis, T1DM, and insulin resistance further complicates metabolic control, increasing the risk of complications like diabetic nephropathy and cardiovascular disease. Biologic treatments targeting IL-17A and IL-17F offer promising therapeutic options for managing both skin and metabolic symptoms. The early identification and management of metabolic risk factors, along with personalized interventions, are essential to improve clinical outcomes in patients with psoriasis and T1DM, particularly in obese individuals. This case report and review highlight the complex interplay of these conditions and emphasize the need for integrated treatment strategies.

## 1. Introduction

Diabetes and psoriasis are systemic diseases widely known to be associated with a high prevalence of obesity, metabolic, and cardiovascular complications. The pathogenesis of both conditions includes an autoinflammatory Th-1-dependent response and abnormal levels of interleukin 17 (IL-17). A proper body weight provides a balance between adipokines and immune cell responsiveness while avoiding hyperactivation. Excessive fat accumulation leads to the activation of immune cells within adipose tissue, which results in chronic low-grade systemic inflammation. This process is particularly relevant in conditions like psoriasis and type 1 diabetes (T1DM), where inflammation aggravates disease outcomes. Here, we present a case of severe psoriasis coexisting with necrobiosis lipoidica in an obese 18-year-old boy suffering from T1DM, and we discuss the common molecular background of the conditions, which, together, drive a vicious circle of metabolic syndrome and cardiovascular burden.

## 2. Case Report

**Figure 1:**
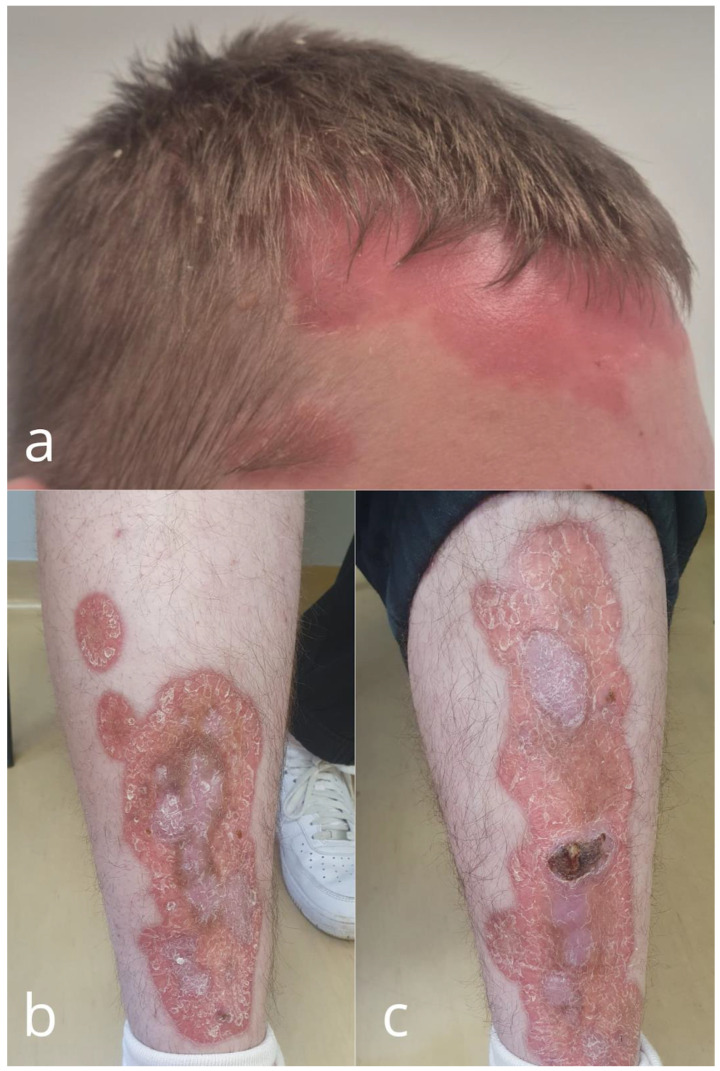
Clinical presentation of the patient: typical psoriatic lesions on the scalp and face, accompanied by erythematous plaques on the lower legs combining clinical features of both psoriasis and necrobiosis lipoidica [8,9]. (**a**) erythematous and desquamative plaques on the scalp and forehead; (**b**) right leg, extensive erythematous plaques with a sharp, slightly elevated border and central atrophy covered with silvery scale; and (**c**) a superficial ulceration in the central part of the plaque on the left leg.

**Figure 2:**
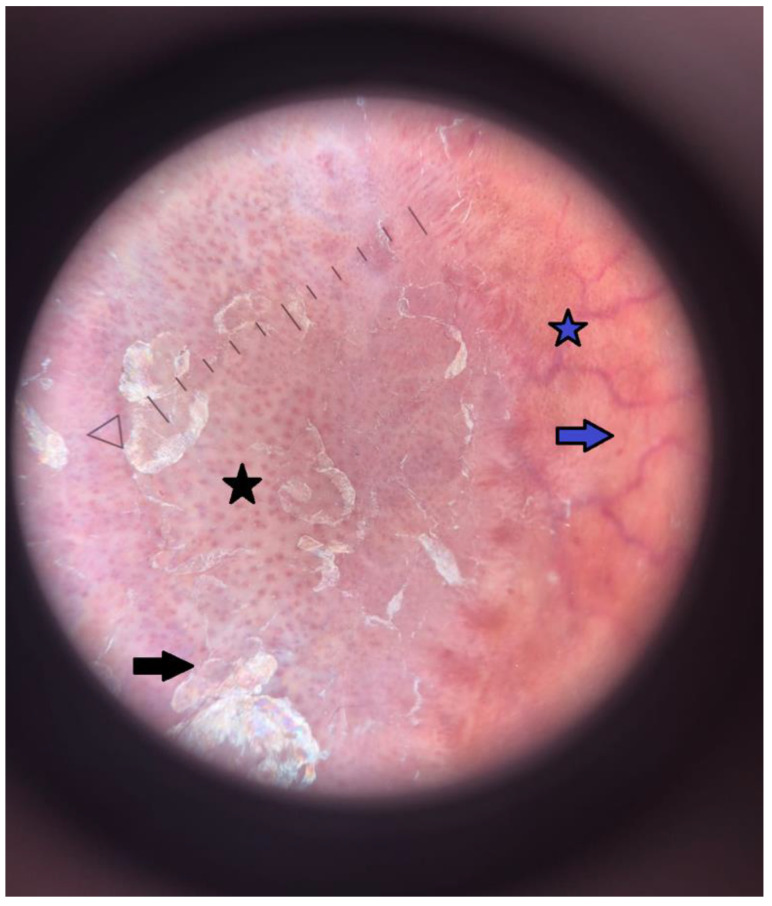
The dermoscopic image of the erythematous plaque on the right shin revealed characteristic patterns of both psoriasis (black) and necrobiosis lipoidica (blue), coexisting within the same skin lesion [10,11]. Black star—regularly distributed dotted vessels with a reddish-pinkish background; black arrow—white scale; blue star—a network of arborizing vessels; blue arrow—orange, homogenous background.

An 18-year-old male with a complex medical history of psoriasis, T1DM, and essential hypertension (HTN) was admitted to the Department of Pediatrics, Endocrinology, Diabetology with Cardiology Divisions for evaluation and to determine a further individualized management strategy. He was first diagnosed with type 1 diabetes mellitus and psoriasis in 2015, at the age of nine. In 2020, essential hypertension was identified, and ramipril at 10 mg once daily was introduced, although adherence remained inconsistent. In February 2022, the patient was hospitalized for control tests and re-education due to persistently elevated HbA1c levels and poorly optimized insulin dosing. On admission, skin examination revealed extensive erythematous and desquamative plaques on the scalp, forehead, trunk, and the anterior parts of the shins (Figure 1). The plaques on the shins were atrophic, and a superficial ulceration on the left leg was observed. Dermoscopy of the lesions revealed an orange background and multiple branching vessels, which led to a diagnosis of necrobiosis lipoidica (NL) coexisting with psoriasis vulgaris (Figure 2). The patient’s metabolic profile was characterized by a height of 187.5 cm, a weight of 123 kg, and a body mass index (BMI) of 34.9 kg/m^2^, placing him above the 97th percentile for his age. His waist circumference measured 115 cm, which exceeds the 97th percentile for 18-year-old males, indicating marked central adiposity. This yielded a waist-to-height ratio (WHtR) of approximately 0.6, placing him in the moderate- to high-risk category for obesity-related cardiometabolic complications [1]. Blood glucose monitoring revealed significant fluctuations, ranging from 90 to 400 mg/dL. His HbA1c level was elevated at 11%, reflecting suboptimal diabetes management and placing him at high risk both for acute and chronic complications of T1DM [2]. His blood pressure was controlled with antihypertensive therapy, recorded at 120/70 mmHg under treatment with ramipril. However, 24-hour ambulatory blood pressure monitoring revealed the following complex pattern: 32.7% of systolic and 2.0% of diastolic blood pressure readings were above reference values during the day. At night, 12.5% of systolic and 0% of diastolic values exceeded reference levels. Notably, a blunted nocturnal dip in blood pressure was observed, with a mean arterial pressure (MAP) reduction of only 6.7%, indicating a suboptimal nocturnal decline in blood pressure [3]. Laboratory analysis revealed triglyceride (TG) levels of 84 mg/dL and HDL cholesterol levels of 64 mg/dL. While this TG level is within normal parameters, the HDL level, although not critically low, is suboptimal for cardiovascular health, particularly in the context of T1DM and obesity [4]. To provide a clearer diagnostic framework, Table 1 summarizes how the patient meets the criteria for metabolic syndrome according to the most commonly used pediatric and adolescent definitions [5,6,7].

Based on the above-mentioned findings, the patient met the criteria for metabolic syndrome (MetS), characterized by central obesity, hypertension managed with antihypertensive therapy, and impaired glucose metabolism evidenced by extreme fluctuations in blood glucose levels (90–400 mg/dL) and an elevated HbA1c of 11%. With regard to psoriasis, the previous treatment with topical corticosteroids and calcipotriol was assessed as inadequate. Due to the overall high cardiovascular risk profile, further treatment with acitretin and cyclosporin A was ruled out. Methotrexate, despite reducing the total cardiovascular risk in psoriatic patients, can impair glucose metabolism. After a thorough evaluation, treatment with an IL-17A/F antagonist, bimecizumab, was prescribed. After 16 weeks of bimekizumab treatment, the patient showed a very good response, with the complete clearance of psoriatic lesions. However, while the necrobiosis lipoidica lesions appeared less inflammatory, the affected area remained stable. Long-term outcomes are still pending further follow-up.

## 3. Discussion

### 3.1. Prevalence of Psoriasis in Type 1 Diabetes

Psoriasis is a chronic autoinflammatory disorder involving skin, nails, and joints that affects about 2–4% of the general population worldwide [12]. It occurs due to an interplay between inherited susceptibility and environmental trigger factors, including intense stress, skin injuries, infections, and certain drugs. The disease is divided into two subtypes based on age of onset and related clinical course. A positive family history, a strong association with human leukocyte antigen Cw6 (HLA-Cw6), and frequent nail and joint involvement constitute distinctive features of early-onset psoriasis (<40 years old) [13]. It is estimated that, in 35% to 50% of cases, the disease begins before the age of 20 [14]. The prevalence of pediatric psoriasis is strictly correlated with age and ranges from 0.37–0.55 in prepubertal children to 1.01–1.37 in adolescents [15,16].

Although an increased risk for psoriasis in individuals with type 1 diabetes was reported in the mid-1980s [17], only a few studies have been aimed at the systemic evaluation of psoriasis incidence in pediatric patients with diabetes. Di Constanzo et al. [18], in an observational study enrolling children with T1DM, reported psoriasis in 9 of the 194 participants (4.7%) compared to 2.1% in the general pediatric population in Italy. Interestingly, psoriasis was more prevalent than vitiligo and alopecia areata, which are traditionally considered to be frequent T1DM skin comorbidities [18]. Similar data were obtained in another single-center observational study conducted at the Pediatric Diabetes Center of Padua University. However, this time, psoriasis prevalence was four times higher in T1DM patients than in healthy controls [19]. Remarkably, the authors indicated that the onset of psoriasis coincided with the onset of diabetes, similar to the presented case. In contrast, a recent Swedish register-based study enrolling 15,188 T1DM patients under the age of 18 and 74,210 healthy controls revealed only a slight but statistically significant difference between the studied subgroups (0.9 vs. 0.7%, respectively) [20]. The risk for psoriasis development was greater in individuals with T1DM (HR = 1.3), and cumulative risk was markedly pronounced after puberty.

### 3.2. Shared Disease Mechanisms

#### 3.2.1. The Key Role of Th17, γδ T, and Th1 Cells in Autoimmune Response

**Figure 3:**
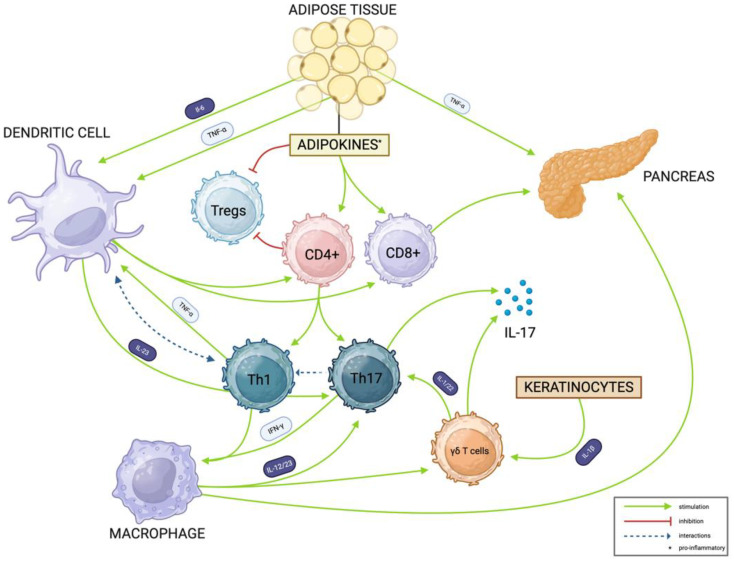
Excessive adipose tissue constitutes major source of proinflammatory cytokines, including TNF-α, IL-6, leptin, and resistin, which promotes naïve CD4+ lymphocyte differentiation toward Th1 and Th17 subtypes. In the initial phase of psoriasis pathogenesis, activated dendritic cells and macrophages release IL-23, which promotes Th17 differentiation. IL-6 and TNF-α released from adipocytes enhance dendritic cells’ interactions with lymphocytes. Moreover, IL-23 together with IL-1β derived from keratinocytes shift cytokine profile produced by γδ T cells towards IL-17A/F. γδ T cells become the main source of IL-17 in autoimmune diseases. Moreover, they produce IL-1 and IL-22, which augment Th17 differentiation and further IL-17 release. IL-17 is responsible for epidermis thickening characteristic of psoriasis and sustaining chronic inflammation seen in psoriasis and T1DM. The role of Th1 cells in psoriasis pathogenesis includes influence on dendritic cells mediated by TNF-α and macrophage activation by IFN-γ. T1DM is initiated by the processing of pancreatic β cell autoantigens by dendritic cells, which then present them in regional lymph nodes to CD4+ lymphocytes. In the presence of co-stimulatory signals and IL-12 (not included in the figure), antigen-specific CD4+ lymphocytes differentiate into Th1 cells that interact de novo with dendritic cells to boost the activation of effector CD8+ cells. In addition, Th1 cells secrete IFN-γ and TNF-α, contributing to the stimulation of inflammation, activation of macrophages, and generation of oxidative stress, consequently leading to β cell apoptosis. Obesity maintains chronic low-grade systemic inflammation and, through leptin, suppresses Tregs function and promotes autoaggressive response. DC—dendritic cell, IL-1β—interleukin 1 beta, IFN-γ—interferon gamma, IL-6—interleukin 6, IL-12—interleukin 12, IL-17—interleukin 17, IL-23—interleukin 23, T1DM—type 1 diabetes mellitus, Th17—T helper 17 cell, TNF-α—tumor necrosis factor alpha, Tregs—regulatory T lymphocytes. Own work based on [8,27,28,29,30,31,32]. Created in BioRender (accessed on 16 April 2025; web-based platform with continuous updates, version not specified). Available online: https://BioRender.com/2tc9bks (accessed on 6 April 2025).

It is widely recognized that autoimmune diseases often coexist, which may result from a common genetic and epigenetic background and exposure to environmental factors. It is noteworthy that up to 20% of patients with an autoimmune disease suffer from one or more autoimmune conditions. The key role in abnormal overactive inflammatory responses may be attributed to Th1 and Th17 signaling, which are involved in the pathogeneses of several autoimmune diseases, e.g., diabetes mellitus, rheumatoid arthritis, and psoriasis [21]. Nowadays, Th17 cells are acknowledged as being pivotal in the pathogenesis of psoriasis, although Th1 cells also play a substantial role in disease development and progression. The cytokine profile of Th1 cells supports the proliferation and differentiation of T lymphocytes, the activation of macrophages, and the enhancement of the production of IL-12 and IL-23, therefore driving Th17-dependent responses [8]. IL-17A is a prototypical member of the IL-17 cytokine family and has been demonstrated to act as a double-edged sword. On the one hand, it mediates the healing response to injury and protective innate immunity against pathogens, especially antifungal protection. On the other hand, it contributes to the development of autoimmunity and the progression of cancer [22,23]. IL-17 is mainly produced by activated memory CD4 T cells of the Th17 subset and, to a lesser extent, by various innate immune cells [24,25]. However, in the case of autoimmune diseases, this proportion changes, and the significant source of IL-17 becomes other immune cells [26]. It has been demonstrated that γδ T cells, a subgroup of innate immune cells that produce IL-17, are involved in both the induction and effector phases of autoimmune inflammation. Even though innate cells cannot trigger an autoimmune response, they might influence the cytokine milieu in a target organ to encourage the development of long-term inflammation. In the physiological state, γδ T cells produce INF-γ and play a role in activating adaptive immune cells. However, due to an environment rich in proinflammatory cytokines, the balance may be turned toward IL-17 and TNF-α secretion, which initiates and maintains an autoaggressive response and boosts inflammation. IL-17 is considered to be the primary mediator of inflammation associated with autoimmune diseases (see Figure 3).

#### 3.2.2. Psoriasis Development

The change in the cytokine production profile by γδ T cells in psoriasis is stimulated by IL-1β ß secreted by keratinocytes and IL-23 produced by macrophages, dendritic cells, and Langerhans cells after stimulation by alloantigens and epidermal autoantigens, including LL37 (antimicrobial peptide derived from keratinocytes) and ADAMTSL5 (protein produced by melanocytes) [33]. Additionally, γδ T cells secrete IL-1 and IL-22, which stimulate the differentiation of Th17 cells, further driving IL-17 production [8]. Another piece of evidence for the fundamental role of γδ T lymphocyte subpopulations in the pathogenesis of psoriasis was provided by a study that demonstrated a greater expression of γδ T cells in psoriatic skin samples compared to healthy controls. Interestingly, γδ T cells were decreased in the sera of the patients, but their levels normalized after effective treatment, which suggests the migration of inflammatory cells from the peripheral blood to the skin with active psoriatic plaques [34]. It has also been shown that γδ T cells possess tissue-resident memory (TRM) properties and, in patients with psoriasis in remission, serve as a source of IL-17 and play a significant role in recruiting inflammatory cells from the peripheral circulation during disease relapse [35]. Moreover, it has been shown that during systemic psoriasis treatment, γδ T cell expression decreases to a lesser degree in the epidermis than in the dermis, which may be related to limited drug penetration. This carries significant clinical implications, emphasizing the necessity of integrating systemic and topical therapies while also extending the duration of the maintenance phase in psoriasis treatment [35,36].

#### 3.2.3. Type 1 Diabetes Mellitus Pathogenesis

T1DM is characterized by the autoimmune progressive destruction of pancreatic ß-cells, mediated mainly by cytotoxic T and innate immune cells [28]. The disease is initiated by the processing of pancreatic β cell autoantigens by dendritic cells, which then present them in regional lymph nodes to CD4+ lymphocytes. In the presence of co-stimulatory signals and IL-12, antigen-specific CD4+ lymphocytes differentiate into Th1 cells that interact de novo with dendritic cells to boost the activation of effector CD8+ cells. In addition, Th1 cells secrete IFN-γ and TNF-α, contributing to the stimulation of inflammation, the activation of macrophages, and the generation of oxidative stress, consequently leading to β cell apoptosis [29].

While Th1 lymphocytes play a definitive role in the effector phase, a growing body of evidence from studies on animal models of diabetes suggests the crucial role of TRM cells, acting through Th17, which may direct the inflammatory response toward Th1 and sustain the recruitment of inflammatory cells from peripheral blood. The presence of TRM cells within pancreatic islets has been confirmed in biopsy specimens taken from individuals with recent-onset type 1 diabetes [37]. In non-obese-diabetic mice, TRM cells have been detected in islets even before the onset of the disease. They constitute the source of C-X-C motif chemokine 10 (CXCL10), which further binds to C-X-C motif chemokine receptor 3 (CXCR3) on cytotoxic T lymphocytes in lymph nodes, thereby triggering the recruitment of cytotoxic T cells into pancreatic islets to enhance insulitis. A recent study proved that the depletion of pancreatic TRM cells, either by fatty acid-binding protein 4 (FABP4) or specific antibodies, reduced inflammation and the onset of diabetes in a mice model [38]. Harmful effects were probably mediated indirectly, and this step was found to be necessary in preclinical studies. An increased number of Th17 cells, which may be converted into Th1 cells [38], was found in the pancreatic lymph nodes of patients with T1DM [39]. Moreover, an increased plasma IL-17 level and raised numbers of circulating IL-17-producing cells and islet antigen-specific Th17 cells were observed in the studied population [40,41]. Nonetheless, certain preceding studies have suggested that IL-17 plays a protective role against T1DM development [42,43]. The source of these contradictory observations seems to be the presence of distinct γδ T cell subpopulations, some of which limit the onset of T1DM, while others aggravate it, potentially through affecting other T cells [44]. Currently, more and more research has focused on developing therapies targeting immunological disturbances at the very early stage of the disease to prevent the development of overt diabetes. Previously mentioned data suggested that targeting Th17 cells, γδ T cells, and IL-17 may constitute an effective causal treatment for T1DM.

### 3.3. Necrobiosis Lipoidica

#### 3.3.1. The Epidemiology of Necrobiosis Lipoidica

Necrobiosis lipoidica is a chronic, inflammatory, granulomatous disease with a poorly understood etiology, originally described as a disorder accompanying diabetes [45]. Between 11% and 87% of individuals with necrobiosis lipoidica are diagnosed with diabetes, whereas only 0.3% to 1.2% of those with diabetes develop NL [46]. The exact prevalence of NL in psoriatic patients is not well-established in large-scale studies. However, it is considered uncommon, but the condition may be more prevalent in patients with psoriasis and concurrent diabetes, particularly T1DM, as both conditions share specific immunological and inflammatory pathways. So far, only four case reports of NL coexisting with psoriasis have been published [47,48,49,50]. In three out of these four cases, diabetes was present, with only one case not exhibiting any glycemic disturbances. However, in the final case, the coexistence of necrobiosis lipoidica and palmoplantar pustulosis was observed rather than plaque psoriasis [50].

#### 3.3.2. Histological Findings in Necrobiosis Lipoidica

Histologically, NL is characterized by the degeneration of collagen and the accumulation of lipids in the skin, especially in the dermis [51]. Although the precise etiology of NL remains unclear, various contributing factors have been identified. The most widely accepted theory suggests the involvement of diabetic microangiopathy, which leads to impaired skin perfusion, necrosis, collagen degradation, and lipid accumulation [9]. Currently, increasing attention is being given to theories regarding immune system dysregulation and the impact of glucose metabolism disturbances on fibroblast function [52]. Immunohistochemical studies have shown that skin samples from NL lesions contain reduced amounts of collagen fibers with pathological cross-linking [53]. Nonetheless, the ratio between collagen type I and type III is correct. Moreover, glucose transporter 1 (GLUT-1) expression in skin fibroblasts is upregulated, which contributes to the development of vascular occlusion and impaired tissue oxygenation [54].

#### 3.3.3. The Immunology of Necrobiosis Lipoidica and Targeted Therapy

Given the fact that insulin resistance and the autoimmune response in pancreatic β-cells have been associated with increased levels of TNF-α, its role in the pathogenesis of NL has also been explored. In detail, TNF-α is a potent activator of macrophages and CD4+ T-helper cells, which are key cells in granulomatous inflammation [51]. It has been suggested that TNF-α is involved in the dysregulation of collagen and extracellular matrix (ECM) remodeling by the activation of matrix metalloproteinases [55]. TNF-α plays a key role in inducing endothelial cell dysfunction by enhancing the prothrombotic state, stimulating the expression of adhesion molecules, and impacting vascular permeability and angiogenesis [56]. Another pathway of macrophage activation is stimulation by IFN-γ produced by Th1 lymphocytes activated by IL-12 [57]. Recent studies have suggested that IL-17 may be elevated in the skin lesions of necrobiosis lipoidica patients [58]. The above reports have formed the basis for attempts to apply biological therapy in necrobiosis lipoidica. To date, the literature primarily contains case series describing the use of various TNF-α inhibitors, IL-12/23 inhibitors (ustekinumab), and IL-17 inhibitors (secukinumab) [59,60,61,62,63]. The response to treatment with biologic drugs is variable, despite the rationale reflected in the disease’s pathogenesis. Generally, the use of ustekinumab and secukinumab is associated with a better clinical response than TNF-α blockers. Improvement in skin lesions was also observed after treatment with IL-12/23 and IL-17 inhibitors in patients who had previously not responded to anti-TNF-α drugs [59,60,61]. These treatment outcomes appear promising; however, in the future, they should be evaluated in studies involving larger patient populations.

### 3.4. Obesity and Autoimmune Disorders

Adipose tissue is an energy reservoir and produces a wide range of bioactive molecules, including adipokines, cytokines, chemokines, and gaseous messengers [64]. A proper body weight provides a balance between adipokines and immune cell responsiveness while avoiding hyperactivation. Adipokines are a crucial link between body fat and overall health. Therefore, they represent a significant focus of research on metabolic health. An excessive accumulation of fat, particularly in cases of diet-induced obesity, leads to the activation of immune cells within adipose tissue, resulting in chronic low-grade systemic inflammation [65]. Therefore, obesity is thought to predispose or aggravate autoimmune diseases. The most studied adipokines include leptin, adiponectin, resistin, TNF-α, and IL-6.

#### 3.4.1. Leptin

Leptin is a hormone primarily produced by adipose tissue that plays a crucial role in regulating energy balance, body weight, and appetite. Leptin is mainly considered to function as an energy sensor by conveying information to the brain regarding the body’s energy storage levels. In response to this signal, the brain initiates appropriate adjustments to modulate food intake and energy expenditure to restore energy homeostasis [66]. However, in addition to its role in metabolism, it is a proinflammatory adipokine, significantly affecting the immune system by influencing innate and adaptive immunity [66,67]. Leptin receptors are expressed on various immune cells—including T cells, B cells, monocytes, and neutrophils—where leptin modulates cytokine production, cell proliferation, and survival [68,69]. In autoimmune pathways, leptin promotes the differentiation and proliferation of Th1 and Th17 cells, which are central to the pathogenesis of autoimmune diseases [30]. Moreover, leptin suppresses the activity of regulatory T cells (Tregs), which is vital for maintaining immune tolerance, thereby exacerbating autoimmune responses [70]. This proinflammatory action of leptin contributes to the imbalance between effector T cells and Tregs observed in autoimmune diseases.

Obesity in children with T1DM contributes to excessive leptin release. However, growing hormone levels lead to a decreased responsiveness of the hypothalamus and inefficient appetite suppression [71]. Another factor influencing the balance between appetite and satiety is insulin, which has been shown to promote leptin secretion and, concurrently, to impair leptin transport across the blood–brain barrier (BBB). Therefore, in the context of insulin therapy in T1DM, it may further raise leptin levels, dysregulate metabolic control, increase appetite, facilitate weight gain, and develop insulin resistance. The above provided the basis for the hypothesis that leptin supplementation can serve as an adjunctive therapy in T1DM and contribute to reducing total insulin requirements and improving lipid metabolism. Metreleptin, a synthetic analogue of the hormone leptin, has been explored for its potential to improve metabolic control in T1DM patients. A clinical trial published in 2017 evaluated the safety and efficacy of metreleptin in patients with suboptimally controlled T1DM [72]. The results showed that while metreleptin did not significantly reduce HbA1c levels, it led to modest decreases in body weight and daily insulin requirements. The study concluded that while metreleptin is safe, it may not be effective in improving glycemic control in T1DM. Until now, metreleptin has been registered for the treatment of congenital and acquired lipodystrophy syndromes to help manage the metabolic complications associated with the conditions.

#### 3.4.2. Resistin

Resistin is a peptide hormone derived from adipose tissue, primarily engaged in the impairment of insulin sensitivity in peripheral tissues, especially muscle [73]. It may also impact the functions of adipocytes themselves, influencing fat storage and the overall metabolic activity of adipose tissue [74]. Resistin is also secreted by specific immune cells, like macrophages, which suggests that it plays a role in immune system regulation [75]. Moreover, it has been demonstrated to augment transcriptional events, which leads to an increased expression of several proinflammatory cytokines, including IL-1, IL-6, IL-12, and TNF-α [76]. Obesity is a known risk factor for psoriasis aggravation. Since resistin is elevated in obesity, it is thought that, throughout the impact on TNF-α and IL-6, resistin can, to a certain extent, explain the association between obesity and the severity of psoriasis. In line with this, observational studies have shown that resistin levels are increased in serum and psoriatic plaques. In addition, this correlates with psoriasis severity and decreases under efficient therapy, including phototherapy and systemic agents [77,78,79]. While resistin’s role in type 2 diabetes is well-documented, there are scarce data on its function in T1DM. Some studies have suggested that resistin levels may be elevated in individuals with T1DM, particularly those with poor glycemic control or higher levels of inflammation. However, Geyikli et al. [80], in their study, which enrolled patients and a control group matched in terms of BMI, established increased serum resistin in diabetic adolescents compared to healthy individuals. Elevated resistin levels may also contribute to the inflammatory burden seen in T1DM and increase the risk of complications such as cardiovascular disease and kidney damage [81,82].

#### 3.4.3. Adiponectin

In contrast to other adipokines, adiponectin has anti-inflammatory and insulin-sensitizing properties, and it is considered to be one of the beneficial adipokines involved in metabolic health [83]. Most studies have demonstrated reduced adiponectin levels in patients with psoriasis compared to healthy controls [84,85,86]. However, studies evaluating the correlation between adiponectin levels and disease severity have presented conflicting results. Most reports have demonstrated that serum adiponectin is negatively correlated with Psoriasis Area and Severity Index (PASI) score [87,88]. However, in the literature, some reports suggest that adiponectin is not related to PASI or that the correlation disappears in patients with severe psoriasis (PASI > 20) [89,90]. In obese individuals, lower adiponectin levels might contribute to an increased inflammatory burden. The relationship between adiponectin and T1DM is still a subject of active research. In type 1 diabetes, adiponectin plays a role in regulating insulin sensitivity and inflammation, both of which are important for managing the disease [91]. Higher adiponectin levels are generally associated with better metabolic control, improved insulin sensitivity, and reduced inflammation, which could help to reduce the risk of complications [92].

#### 3.4.4. IL-18

IL-18 is a proinflammatory cytokine produced constitutively in biologically inactive preform, mainly by macrophages and other immune cells, and activated by caspase-1 in response to lipopolysaccharides [93]. Accumulating data have demonstrated a novel alternative pathway of IL-18 activation in a caspase-independent manner, which occurs in the epithelium and adipose tissue [94,95]. IL-18 promotes the activation and proliferation of T cells, particularly Th1 and Th17 cells, which are known to play central roles in the pathogeneses of psoriasis and T1DM [96,97,98]. IL-18 can drive the release of IL-17 and other cytokines in the skin, which contributes to the thickening of the epidermis and the scaling characteristic of psoriatic lesions [31]. Research conducted by Ohta et al. demonstrated that serum IL-18 levels are elevated in psoriatic skin samples [99]. Moreover, it was disclosed that the levels of IL-18 are raised in active psoriatic plaques compared to those observed in stable disease [100]. The Il-18 serum concentration is also elevated in psoriatic patients compared to healthy individuals [101,102,103].

In T1DM, IL-18 has been implicated in the development and progression of the disease, particularly in the autoimmune destruction of pancreatic β-cells [104]. Interestingly, IL-18 and T1DM share some genetic regions, specifically regarding genetic susceptibility [105]. Research has shown that variations in genes related to the immune system, including the IL-18 gene, can contribute to an increased risk of developing T1DM. Several studies have found elevated levels of IL-18 in both the blood and the pancreatic islets of individuals with T1DM, suggesting that it plays a role in the inflammatory process that leads to beta cell loss [106]. IL-18 may contribute to this by enhancing the activation of Th1 and influencing the function of immune cells like T cells, NK cells, and macrophages, potentially amplifying the immune attack on pancreatic β-cells. Some animal studies have shown that blocking IL-18 can help to reduce the autoimmune response and prevent or delay the onset of diabetes [32]. It is a promising target for future therapies aimed at modulating the immune system in T1DM and psoriasis, though more research is required to determine its full therapeutic potential.

With regard to metabolic syndrome, elevated IL-18 levels can contribute to insulin resistance by increasing the production of other inflammatory cytokines like TNF-α and IL-6, which interfere with insulin signaling pathways [107]. Despite IL-18 not being a routine biomarker for clinical diagnosis, its role in the inflammation driving metabolic syndrome suggests potential implications for understanding and treating the condition.

### 3.5. Double Diabetes

#### 3.5.1. Coincidence of Insulin Resistance in Autoimmune-Mediated Diabetes

Nowadays, the traditional classification into type 1 and type 2 diabetes is not obvious due to the obesity pandemic and the increasing co-existence of the autoinflammatory process and insulin resistance. Several terms in the literature describe this condition, including double diabetes, hybrid diabetes, and type 1.5 [108]. Of note, this population is more prone to the development of diabetic nephropathy and cardiovascular complications [109], which creates an urgent need for new strategies for the optimal management of these patients. Unfortunately, precise data on the prevalence of insulin resistance among patients with T1DM have not been established. An Australian study conducted on a population of 2120 adults with T1DM estimated the prevalence of metabolic syndrome at 30%, with this prevalence increasing with age and being associated with a higher incidence of macrovascular and microvascular complications [110]. With regard to the pediatric population, a small study conducted in Denmark showed that metabolic syndrome occurred more frequently in children with T1DM compared to their healthy peers [111]. Furthermore, among all children diagnosed with metabolic syndrome, those with coexisting T1DM had a lower BMI and a smaller waist circumference. In line with this, Castro-Correira et al. emphasized that MetS risk factors should be routinely assessed in all diabetic adolescents, including those with a healthy BMI [112].

#### 3.5.2. Genetic Susceptibility and Molecular Mechanisms

The mechanisms underlying the development of insulin resistance in patients with T1DM have not yet been fully elucidated. Certainly, the risk for insulin resistance is mainly attributed to an individual’s genetic predisposition related to HLA genes and lifestyle habits [113]. The accumulation of adipose tissue results in the production of several hormones and the release of free fatty acids into the bloodstream, becoming the main energy source. Then, the glucose serum level rises, and a higher insulin dosage is needed. Difficulties in maintaining a balance in intensive insulin therapy and the fear of hypoglycemia are also mentioned as some of the reasons for weight gain.

A complex network of interactions between the cytokines produced by adipose tissue and the hormones secreted by the pancreas is involved in regulating metabolism [114]. Adipokines are biologically active signaling molecules that constitute a link between the endocrine and immune systems. Most of them are positively correlated with overall fat mass and are involved in pancreatic β-cell dysfunction and insulin resistance. The exception is adiponectin, whose level is inversely correlated with adipose tissue mass [115]. Adiponectin protects β-cells from glucotoxicity-induced apoptosis and dysfunction [116], whereas the main pro-inflammatory adipokines, leptin and resistin, were found to exacerbate pancreatic β-cells apoptosis [117,118].

Moreover, as mentioned before, T1DM is a state of IL-17 overproduction [40,41]. An increased level of IL-17 activates an overabundant proinflammatory response by NF-kB signaling in adipose tissue, which results in the excessive production of IL-1β, IL-6, and TNF-α, contributing to insulin resistance [119]. Taken together, individuals genetically predisposed to insulin resistance with accompanying autoimmune disorders have a cumulatively higher risk of developing diabetes.

### 3.6. Psoriasis, Obesity, and Insulin Resistance

#### 3.6.1. Bidirectional Interaction Between Adipose Tissue Level and Psoriasis Severity

Obesity and metabolic syndrome are notably more prevalent among individuals with psoriasis, suggesting a bidirectional relationship where each condition may exacerbate the other. For instance, Gisondi et al. [120] reported that obesity was more prevalent among psoriasis patients, with 30% of psoriasis patients being obese compared to 20% of control subjects without psoriasis, indicating an association between psoriasis and an increased risk of obesity. Carrascosa et al. [121] similarly observed a correlation, reporting that individuals with psoriasis exhibited a higher likelihood of obesity, quantified by an odds ratio (OR) of 1.8, thus reinforcing the link between these two conditions. Armstrong et al.’s meta-analysis [122] corroborated these observations, demonstrating that individuals with psoriasis exhibited a significantly elevated likelihood of obesity (OR: 1.66; 95% CI: 1.46–1.89).

As it turns out, an increased BMI elevates the risk of developing psoriasis and correlates with a greater disease severity. Naldi et al. [123] demonstrated a dose-dependent relationship between a higher BMI and an increased psoriasis severity measured by the PASI, indicating that psoriasis tends to be more severe as BMI increases. Herron et al. [124] similarly found that patients with obesity are more likely to have severe psoriasis, with higher PASI scores compared to non-obese patients, suggesting that obesity may exacerbate the clinical manifestations of psoriasis. In a prospective cohort study, Setty et al. [125] observed that women with a BMI of ≥35 kg/m^2^ had a 2.69-fold increased risk of developing psoriasis compared to those with a BMI between 21 and 23 kg/m^2^. These findings suggest that obesity coexists with psoriasis and may contribute to its development and progression.

#### 3.6.2. Psoriasis Increases the Risk for Insulin Resistance

Epidemiological studies have demonstrated a significant association between psoriasis and insulin resistance. Armstrong et al. [126] conducted a systematic review and meta-analysis, revealing that patients with psoriasis have a higher prevalence of insulin resistance and MetS than the general population. Similarly, Langan et al. [27] found that individuals with psoriasis were more likely to develop type 2 diabetes mellitus (T2DM), with the risk increasing alongside the severity of psoriasis. Furthermore, a study by Gyldenløve et al. [127] using hyperinsulinemic-euglycemic clamp techniques confirmed that patients with psoriasis have a reduced insulin sensitivity, even without overt glucose intolerance.

Psoriasis is associated with systemic inflammation characterized by elevated levels of pro-inflammatory cytokines such as TNF-α and IL-6, which not only contribute to skin pathology, but also interfere with metabolic processes [128]. Adipokines derived from excessive adipose tissue further promote inflammation and insulin resistance [129]. Due to this interconnectedness, it is crucial to recognize psoriasis as more than just a skin disease; it also has notable effects on metabolism.

Genetic predisposition is crucial in developing psoriasis and insulin resistance, indicating overlapping metabolic and autoimmune mechanisms [130]. Specific genetic loci, such as those associated with the interleukin-23 (IL-23)/Th17 axis, are implicated in the pathogenesis of psoriasis and are also involved in metabolic regulation and insulin sensitivity [126]. Shared genetic variants affecting T-cell function and cytokine signaling pathways contribute to autoimmune responses and metabolic dysfunction [131]. The chronic inflammation inherent in psoriasis leads to the activation of immune cells and the release of cytokines that disrupt insulin signaling pathways, mainly through the serine phosphorylation of insulin receptor substrate-1 (IRS-1), thereby promoting insulin resistance [132].

#### 3.6.3. Leptin as a Molecular Link Between Obesity, Psoriasis, and Metabolic Syndrome

A growing body of evidence suggests that leptin may be a molecular link connecting obesity, psoriasis severity, and metabolic comorbidities. Leptin enhances Th17-mediated inflammation by promoting the differentiation of naïve T cells into Th17 cells, leading to an increased production of IL-17, a cytokine pivotal in psoriasis pathogenesis [133]. Similarly, leptin enhances keratinocyte proliferation and cytokine production, exacerbating psoriatic lesion development symptoms [134]. Multiple studies have consistently demonstrated that serum leptin levels are significantly elevated in patients with psoriasis and positively correlate with disease severity. Baran et al. [89] found that leptin levels were higher in psoriasis patients and were correlated with PASI, independently of BMI. Similarly, Hwang et al. [135] and Dopytalska et al. [136] observed that increased leptin levels were associated with a greater psoriasis severity, suggesting leptin as a potential biomarker for disease activity. Chen et al. [135] further demonstrated that psoriasis patients exhibit significantly elevated serum leptin levels compared to healthy controls, and these levels are associated with the metabolic disturbances characteristic of the disease, such as insulin resistance and dyslipidemia. This observation reinforces the association between leptin and psoriatic inflammation. These findings suggest that leptin reflects adipose tissue mass and actively participates in the inflammatory processes underlying psoriasis pathogenesis.

#### 3.6.4. The Impact of the Disease Burden on Patient Care Strategy and Treatment Outcomes

Clinically, the coexistence of psoriasis and insulin resistance has considerable implications. Insulin resistance not only complicates the management of psoriasis, but also elevates the risk of cardiovascular diseases and T2DM, contributing to an increased morbidity and mortality [16]. The pro-inflammatory state in psoriasis may accelerate atherosclerosis, and insulin resistance further exacerbates endothelial dysfunction [137]. Recognizing and addressing insulin resistance in patients with psoriasis is, therefore, essential. Early identification allows for lifestyle interventions targeting weight reduction and metabolic control, which can ameliorate cutaneous and systemic symptoms [138]. Therapeutic interventions targeting inflammatory pathways have the potential to enhance insulin sensitivity and mitigate cardiovascular risks, underscoring the importance of holistic management strategies for individuals with psoriasis [139].

Epidemiological data also highlight the impact of obesity on psoriasis treatment and outcomes. Obesity may diminish the effectiveness of systemic therapies, including biologic agents such as TNF-α inhibitors. Carrascosa et al. [121] noted that obese patients may have a reduced response to treatments like TNF-α inhibitors and may require higher doses. The diminished effectiveness observed is attributable to multiple underlying mechanisms. Notably, adipose tissue, particularly visceral fat, functions as an endocrine organ, releasing pro-inflammatory cytokines such as TNF-α, IL-6, and leptin. This secretion contributes to a sustained, low-grade inflammatory condition [140]. In obese patients, elevated levels of TNF-α may diminish the efficacy of standard doses of TNF-α inhibitors, necessitating higher or weight-adjusted dosing to achieve optimal therapeutic outcomes. Obesity affects how biological agents are processed by the body, altering drug distribution and metabolism. This can result in lower concentrations of fixed-dose medications, like specific TNF-α inhibitors, in the bloodstream, ultimately diminishing their therapeutic efficacy [121]. Therefore, employing weight-based dosing approaches could optimize therapeutic outcomes for obese individuals affected by psoriasis.

Conversely, weight reduction has been shown to improve psoriasis severity and enhance treatment responses. Gisondi et al. [120] conducted a randomized controlled trial and demonstrated that a low-calorie diet resulting in weight loss significantly improved PASI scores among obese patients undergoing systemic therapy. A meta-analysis by Upala et al. [141] further indicated that weight loss interventions are associated with significant reductions in psoriasis severity and an improved quality of life. These results indicate that interventions targeting obesity can diminish inflammation linked to excessive adipose tissue. Furthermore, such interventions may improve the effectiveness of systemic treatments by refining pharmacokinetic parameters and alleviating general inflammation.

### 3.7. Metabolic Syndrome in Psoriatic Population

#### 3.7.1. Definition of Metabolic Syndrome

Metabolic syndrome is a cluster of interconnected risk factors that elevate the risk of developing cardiovascular disease (CVD), type 2 diabetes, and other serious health issues. MetS in children is defined by the presence of central obesity, indicated by a waist circumference at or above the 90th percentile (or adult cut-offs if lower), along with at least two of the following criteria: hypertension (systolic BP of ≥130 mmHg, diastolic BP of ≥85 mmHg, or treatment with antihypertensive medication), hypertriglyceridemia (TG of ≥150 mg/dL), low HDL cholesterol (HDL of <40 mg/dL), and impaired glucose regulation (fasting plasma glucose of ≥100 mg/dL or a diagnosis of type 2 diabetes) [142]. MetS in pediatric populations is closely associated with insulin resistance, which plays a central role in the dysregulation of adipose tissue distribution and subsequent metabolic derangements [143]. Contributory environmental influences, including hypercaloric dietary patterns, sedentary behavior, and inadequate sleep, are aggravating factors, frequently intensifying underlying genetic susceptibilities [144]. Unlike adults, defining MetS in children is complicated by physiological changes during growth and puberty and variations based on sex and ethnicity [145].

#### 3.7.2. Patient Care Strategy to Reduce Metabolic and Cardiovascular Risk

The association between psoriasis, obesity, and metabolic syndrome has been shown to have several clinical implications. Psoriasis patients have a higher prevalence of metabolic syndrome components, including obesity, hypertension, dyslipidemia, and insulin resistance, compared to the general population [136]. This combination synergistically increases the risk for CVD; for example, Gelfand et al. [146] found that severe psoriasis is associated with a 1.5-fold increased risk of myocardial infarction. Studies suggest that children with psoriasis, especially those with moderate-to-severe disease, exhibit a higher prevalence of MetS compared to their healthy peers [147,148,149]. The early identification and management of metabolic risk factors in these patients are essential to reduce the likelihood of developing conditions like T2DM and heart disease. Lifestyle interventions such as weight management, physical activity, and dietary modifications are critical to managing psoriasis and metabolic risk factors.

Increased cardiovascular risk should be considered when selecting systemic therapy for psoriasis. Conventional pharmacotherapy is associated with numerous side effects, including elevated levels of total cholesterol, triglycerides, blood glucose, and uric acid, which can negatively impact metabolic health [150]. Biologic drugs have different mechanisms of action, and, except for anti-TNF-α agents, which depress cardiac muscle function and are contraindicated in advanced heart failure, they benefit overall cardiovascular risk [151]. The beneficial effects of specific treatments may stem from their capacity to suppress pro-inflammatory cytokines, key players in the development of MetS and psoriasis. By reducing cytokine production, these treatments could alleviate symptoms in both conditions. To improve patient well-being, healthcare providers should carefully assess and manage metabolic risk factors in psoriasis patients. Combining dermatological and metabolic care is crucial for a complete and effective treatment strategy.

#### 3.7.3. The Rationale for Choosing Anti IL-17A/F Therapy

In the presented case, the dermatological treatment consisted of topical agents with the human monoclonal antibody bimekizumab, targeting IL-17A, IL-17F, and IL-17AF cytokines. IL-17F shares a high degree of homology (55%) with IL-17A. They bind to the same receptors and exhibit similar biological functions [152]. According to clinical trials [153], the dual neutralization of IL-17A and IL-17F provides a more prominent suppression of inflammation, resulting in a more significant improvement in clinical outcomes in psoriasis than IL-17A inhibitors. In the BE RADIANT trial, bimekizumab demonstrated a superior efficacy compared to secukinumab in achieving complete skin clearance (PASI 100) at both week 16 (61.7% vs. 48.9%) and week 48 (67.0% vs. 46.2%) in patients with moderate-to-severe plaque psoriasis [154]. Additionally, the BE VIVID trial reported that bimekizumab achieved greater skin clearance than ustekinumab at week 16, with 85% of patients reaching PASI 90 compared to 50% in the ustekinumab group. This superior efficacy was maintained through week 52 [153]. Moreover, as IL-17A and IL-17F influence vascular endothelial cells, they may contribute to the microvascular changes observed in NL and diabetic microangiopathy. Beyond its effects on endothelial function, inhibiting IL-17A/F may also diminish inflammation, using innovative therapeutic possibilities, notably for diabetic microvascular conditions such as retinopathy. Nevertheless, this perspective is currently theoretical, necessitating empirical validation through prospective scientific investigations to substantiate these hypotheses.

### 3.8. The Significance of Interdisciplinary Care for Patients with Multimorbidity

An additional challenge in adolescence is the psychosocial burden associated with managing multiple chronic diseases. This developmental stage is already marked by significant emotional, social, and psychological changes, and the presence of chronic illness can further complicate the transition to adulthood. Adolescents managing multiple chronic diseases face a heightened risk of developing anxiety and depression, which can adversely affect their social interactions and self-esteem. The relationship between chronic illness and mental health is bidirectional; chronic physical conditions can contribute to the development of mental health disorders, while psychological distress may exacerbate physical disease symptoms. To address these challenges, implementing a multidisciplinary care approach is essential. Integrating psychological interventions, such as cognitive–behavioral therapy, has been shown to improve coping strategies and reduce symptoms of anxiety and depression in adolescents with chronic illnesses [155]. Additionally, in the presented case, dietary recommendations would not only support overall health, but also play a crucial role in managing diabetes by helping to regulate blood glucose levels, reduce inflammation, and improve energy balance. A well-structured nutritional plan, combined with psychological support, may enhance both physical and mental well-being, contributing to better disease control and quality of life. By integrating psychological support and nutritional counseling into standard care, healthcare providers can offer more comprehensive support, potentially improving treatment adherence and enhancing the overall quality of life for adolescents managing multiple chronic conditions.

## 4. Conclusions

In summary, the described case presents an adolescent patient with an extremely high cardiovascular risk, resulting from the coexistence of obesity, diabetes, and psoriasis, which, through a complex network of cytokine interactions, drive systemic inflammation and negatively affect each other. The coexistence of necrobiosis lipoidica appears to be another indicator of metabolic disturbances. Given the patient’s young age, the presence of significant metabolic disorders may lead to adverse cardiovascular events at an early age and contribute to a reduction in overall life expectancy. In this case, a holistic approach is crucial, ensuring not only appropriate pharmacological treatment with minimal metabolic consequences, but also the implementation of healthy dietary habits, regular physical activity, and psychological support. The treatment proposed includes bimekizumab, an IL-17 inhibitor, which has a proven high efficacy in psoriasis, along with a favorable cardiovascular profile and potentially beneficial effects on the course of diabetes and necrobiosis lipoidica, based on available molecular studies and case reports.

## Figures and Tables

**Table 1:** Diagnostic Criteria for Metabolic Syndrome in Adolescents and Adults according to Cook et al. and de Ferranti et al. [5,6] and the International Diabetes Federation (IDF).

Criterion	Cook et al. [5]	de Ferranti et al. [6]	IDF Adult Criteria (≥16 Year)
Waist Circumference	>90th percentile for age/sex	≥75th percentile for age/sex	WC ≥ 94 cm (men)
Triglycerides	≥110 mg/dL	≥100 mg/dL	≥150 mg/dL
HDL Cholesterol	≤40 mg/dL	<50 mg/dL	<40 mg/dL (men)
Blood Pressure	Systolic or diastolic BP ≥ 90th percentile for age, sex, height	≥90th percentile for age, sex, height	≥130/85 mmHg
Glucose/Insulin	Fasting glucose ≥ 110 mg/dL (some use ≥ 100 mg/dL)	Fasting glucose ≥ 110 mg/dL (some use ≥ 100 mg/dL)	Fasting glucose ≥ 100 mg/dL
Number of Abnormal Factors	≥3 of the above	≥2 of the above + elevated LDL or total cholesterol can also be considered in some versions	Central obesity and ≥2 of the remaining factors
Age Range	Children and adolescents (~8–19 yrs)	Children and adolescents (~8–19 yrs)	Adults ≥ 16 yr (IDF recommends adult thresholds from age 16 onward)

## Data Availability

No new data were created or analyzed in this study.

## Abbreviations

The following abbreviations are used in this manuscript:

ABPM	Ambulatory Blood Pressure Monitoring
ADAMTSL	Protein Produced by Melanocytes
BBB	Blood–Brain Barrier
BMI	Body Mass Index
BP	Blood Pressure
CD	Cluster of Differentiation
CXCL10	C-X-C motif chemokine 10
CXCR3	C-X-C motif chemokine receptor 3
ECM	Extracellular Matrix
FABP4	Fatty Acid-Binding Protein 4
GLUT-1	Glucose Transporter 1
HLA	Human Leukocyte Antigen
HR	Hazard Ratio
HTN	Hypertension
LL37	Antimicrobial Peptide Derived from Keratinocytes
MAP	Mean Arterial Pressure
MetS	Metabolic Syndrome
NL	Necrobiosis Lipoidica
NK cells	Natural Killer Cells
OR	Odds Ratio
PASI	Psoriasis Area and Severity Index
T1DM	Type 1 Diabetes Mellitus
T2DM	Type 2 Diabetes Mellitus
TG	Triglycerides
Th1	T Helper Type 1 Cells
Th17	T Helper Type 17 Cells
TNF-α	Tumor Necrosis Factor Alpha
Tregs	Regulatory T Cells
TRM	Tissue-Resident Memory Cells
WHtR	Waist-to-Height Ratio
γδ T cells	Gamma Delta T Cells

## References

[1] Ukegbu T.E., Wylie-Rosett J., Groisman-Perelstein A.E., Diamantis P.M., Rieder J., Ginsberg M., Lichtenstein A.H., Matthan N.R., Shankar V. (2023). Waist-to-Height Ratio Associated Cardiometabolic Risk Phenotype in Children with Overweight/Obesity. BMC Public Health.

[2] Cho S.H., Kim S., Lee Y.-B., Jin S.-M., Hur K.Y., Kim G., Kim J.H. (2023). Impact of Continuous Glucose Monitoring on Glycemic Control and Its Derived Metrics in Type 1 Diabetes: A Longitudinal Study. Front. Endocrinol..

[3] de la Sierra A., Staplin N., Ruilope L.M., Gorostidi M., Vinyoles E., Segura J., Baigent C., Williams B. (2024). A Blunted Nocturnal Blood Pressure Decline Is Associated with All-Cause and Cardiovascular Mortality. J. Hypertens..

[4] Lee J.S., Chang P.-Y., Zhang Y., Kizer J.R., Best L.G., Howard B.V. (2017). Triglyceride and HDL-C Dyslipidemia and Risks of Coronary Heart Disease and Ischemic Stroke by Glycemic Dysregulation Status: The Strong Heart Study. Diabetes Care.

[5] Cook S., Weitzman M., Auinger P., Nguyen M., Dietz W.H. (2003). Prevalence of a Metabolic Syndrome Phenotype in Adolescents: Findings From the Third National Health and Nutrition Examination Survey, 1988–1994. Arch. Pediatr. Adolesc. Med..

[6] de Ferranti S.D., Gauvreau K., Ludwig D.S., Neufeld E.J., Newburger J.W., Rifai N. (2004). Prevalence of the Metabolic Syndrome in American Adolescents: Findings from the Third National Health and Nutrition Examination Survey. Circulation.

[7] Kassi E., Pervanidou P., Kaltsas G., Chrousos G. (2011). Metabolic Syndrome: Definitions and Controversies. BMC Med..

[8] Samotij D., Nedoszytko B., Bartosińska J., Batycka-Baran A., Czajkowski R., Dobrucki I.T., Dobrucki L.W., Górecka-Sokołowska M., Janaszak-Jasienicka A., Krasowska D. (2020). Pathogenesis of Psoriasis in the “Omic” Era. Part I. Epidemiology, Clinical Manifestation, Immunological and Neuroendocrine Disturbances. Adv. Dermatol. Allergol. Dermatol. Alergol..

[9] Reid S.D., Ladizinski B., Lee K., Baibergenova A., Alavi A. (2013). Update on Necrobiosis Lipoidica: A Review of Etiology, Diagnosis, and Treatment Options. J. Am. Acad. Dermatol..

[10] Errichetti E., Ioannides D., Lallas A. (2023). Psoriasis [Łuszczyca]. Dermoscopy in General Dermatology [Dermatoskopia w dermatologii ogólnej].

[11] Errichetti E., Ioannides D., Lallas A. (2023). Necrobiosis lipoidica [Obumieranie tłuszczowate]. Dermoscopy in General Dermatology [Dermatoskopia w dermatologii ogólnej].

[12] Parisi R., Symmons D.P.M., Griffiths C.E.M., Ashcroft D.M. (2013). Identification and Management of Psoriasis and Associated ComorbidiTy (IMPACT) project team Global Epidemiology of Psoriasis: A Systematic Review of Incidence and Prevalence. J. Investig. Dermatol..

[13] Fatema F., Ghoshal L., Saha A., Agarwal S., Bandyopadhyay D. (2021). Early-Onset Versus Late-Onset Psoriasis: A Comparative Study of Clinical Variables, Comorbidities, and Association with HLA CW6 in a Tertiary Care Center. Indian J. Dermatol..

[14] Raychaudhuri S.P., Gross J. (2000). A Comparative Study of Pediatric Onset Psoriasis with Adult Onset Psoriasis. Pediatr. Dermatol..

[15] Augustin M., Glaeske G., Radtke M.A., Christophers E., Reich K., Schäfer I. (2010). Epidemiology and Comorbidity of Psoriasis in Children. Br. J. Dermatol..

[16] Gelfand J.M., Weinstein R., Porter S.B., Neimann A.L., Berlin J.A., Margolis D.J. (2005). Prevalence and Treatment of Psoriasis in the United Kingdom: A Population-Based Study. Arch. Dermatol..

[17] Montagnani A., Tosti A., Patrizi A., Salardi S., Cacciari E. (1985). Diabetes Mellitus and Skin Diseases in Childhood. Dermatologica.

[18] Di Costanzo L., Fattorusso V., Mozzillo E., Patrì A., Di Caprio R., De Nitto E., Balato N., Franzese A. (2017). Psoriasis in Children with Type 1 Diabetes: A New Comorbidity to Be Considered?. Acta Diabetol..

[19] Caroppo F., Galderisi A., Moretti C., Ventura L., Belloni Fortina A. (2021). Prevalence of Psoriasis in a Cohort of Children and Adolescents with Type 1 Diabetes. J. Eur. Acad. Dermatol. Venereol. JEADV.

[20] Samuelsson J., Bertilsson R., Bülow E., Carlsson S., Åkesson S., Eliasson B., Hanas R., Åkesson K. (2024). Autoimmune Comorbidity in Type 1 Diabetes and Its Association with Metabolic Control and Mortality Risk in Young People: A Population-Based Study. Diabetologia.

[21] McGeachy M.J., Cua D.J., Gaffen S.L. (2019). The IL-17 Family of Cytokines in Health and Disease. Immunity.

[22] Amatya N., Garg A.V., Gaffen S.L. (2017). IL-17 Signaling: The Yin and the Yang. Trends Immunol..

[23] Sparber F., LeibundGut-Landmann S. (2019). Interleukin-17 in Antifungal Immunity. Pathogens.

[24] Albanesi C., Cavani A., Girolomoni G. (1999). IL-17 Is Produced by Nickel-Specific T Lymphocytes and Regulates ICAM-1 Expression and Chemokine Production in Human Keratinocytes: Synergistic or Antagonist Effects with IFN-Gamma and TNF-Alpha. J. Immunol..

[25] Cua D.J., Tato C.M. (2010). Innate IL-17-Producing Cells: The Sentinels of the Immune System. Nat. Rev. Immunol..

[26] Ogawa E., Sato Y., Minagawa A., Okuyama R. (2018). Pathogenesis of Psoriasis and Development of Treatment. J. Dermatol..

[27] Reis B.S., Lee K., Fanok M.H., Mascaraque C., Amoury M., Cohn L., Rogoz A., Dallner O.S., Moraes-Vieira P.M., Domingos A.I. (2015). Leptin Receptor Signaling in T Cells Is Required for Th17 Differentiation. J. Immunol..

[28] Lehuen A., Diana J., Zaccone P., Cooke A. (2010). Immune Cell Crosstalk in Type 1 Diabetes. Nat. Rev. Immunol..

[29] Burrack A.L., Martinov T., Fife B.T. (2017). T Cell-Mediated Beta Cell Destruction: Autoimmunity and Alloimmunity in the Context of Type 1 Diabetes. Front. Endocrinol..

[30] Procaccini C., La Rocca C., Carbone F., De Rosa V., Galgani M., Matarese G. (2017). Leptin as Immune Mediator: Interaction between Neuroendocrine and Immune System. Dev. Comp. Immunol..

[31] Shimoura N., Nagai H., Fujiwara S., Jimbo H., Yoshimoto T., Nishigori C. (2017). Interleukin (IL)-18, Cooperatively with IL-23, Induces Prominent Inflammation and Enhances Psoriasis-like Epidermal Hyperplasia. Arch. Dermatol. Res..

[32] Zaccone P., Phillips J., Conget I., Cooke A., Nicoletti F. (2005). IL-18 Binding Protein Fusion Construct Delays the Development of Diabetes in Adoptive Transfer and Cyclophosphamide-Induced Diabetes in NOD Mouse. Clin. Immunol..

[33] Cai Y., Shen X., Ding C., Qi C., Li K., Li X., Jala V.R., Zhang H., Wang T., Zheng J. (2011). Pivotal Role of Dermal IL-17-Producing Γδ T Cells in Skin Inflammation. Immunity.

[34] Qi C., Wang Y., Li P., Zhao J. (2021). Gamma Delta T Cells and Their Pathogenic Role in Psoriasis. Front. Immunol..

[35] Owczarczyk Saczonek A., Krajewska-Włodarczyk M., Kasprowicz-Furmańczyk M., Placek W. (2020). Immunological Memory of Psoriatic Lesions. Int. J. Mol. Sci..

[36] Owczarczyk-Saczonek A., Kasprowicz-Furmańczyk M., Czerwińska J., Krajewska-Włodarczyk M., Placek W. (2022). The Effect of Therapy on TRM in Psoriatic Lesions. Postep. Dermatol. Alergol..

[37] Kuric E., Seiron P., Krogvold L., Edwin B., Buanes T., Hanssen K.F., Skog O., Dahl-Jørgensen K., Korsgren O. (2017). Demonstration of Tissue Resident Memory CD8 T Cells in Insulitic Lesions in Adult Patients with Recent-Onset Type 1 Diabetes. Am. J. Pathol..

[38] Wu X., Cheong L.Y., Yuan L., Jin L., Zhang Z., Xiao Y., Zhou Z., Xu A., Hoo R.L., Shu L. (2024). Islet-Resident Memory T Cells Orchestrate the Immunopathogenesis of Type 1 Diabetes through the FABP4-CXCL10 Axis. Adv. Sci..

[39] Haskins K., Cooke A. (2011). CD4 T Cells and Their Antigens in the Pathogenesis of Autoimmune Diabetes. Curr. Opin. Immunol..

[40] Marwaha A.K., Leung N.J., McMurchy A.N., Levings M.K. (2012). TH17 Cells in Autoimmunity and Immunodeficiency: Protective or Pathogenic?. Front. Immunol..

[41] Reinert-Hartwall L., Honkanen J., Salo H.M., Nieminen J.K., Luopajärvi K., Härkönen T., Veijola R., Simell O., Ilonen J., Peet A. (2015). Th1/Th17 Plasticity Is a Marker of Advanced β Cell Autoimmunity and Impaired Glucose Tolerance in Humans. J. Immunol..

[42] Bellemore S.M., Nikoopour E., Schwartz J.A., Krougly O., Lee-Chan E., Singh B. (2015). Preventative Role of Interleukin-17 Producing Regulatory T Helper Type 17 (Treg 17) Cells in Type 1 Diabetes in Non-Obese Diabetic Mice. Clin. Exp. Immunol..

[43] Lau K., Benitez P., Ardissone A., Wilson T.D., Collins E.L., Lorca G., Li N., Sankar D., Wasserfall C., Neu J. (2011). Inhibition of Type 1 Diabetes Correlated to a Lactobacillus Johnsonii N6.2-Mediated Th17 Bias. J. Immunol..

[44] O’Brien R.L., Matsuda J., Aydintug M.K., Jin N., Phalke S., Born W.K. (2022). A Distinctive Γδ T Cell Repertoire in NOD Mice Weakens Immune Regulation and Favors Diabetic Disease. Biomolecules.

[45] Körber A., Dissemond J. (2007). Necrobiosis Lipoidica Diabeticorum. CMAJ Can. Med. Assoc. J..

[46] Peyrí J., Moreno A., Marcoval J. (2007). Necrobiosis Lipoidica. Semin. Cutan. Med. Surg..

[47] Dibanian R.S., Muradian A.G. (1982). Association of psoriasis with sarcoid-like form of necrobiosis lipoidica. Vestn. Dermatol. Venerol..

[48] Ito H., Imamura S. (2016). Scald-Induced Necrobiosis Lipoidica in a Patient with Diabetes Mellitus and Psoriasis. Case Rep. Dermatol..

[49] Abraham Z., Lahat N., Kinarty A., Feuerman E.J. (1990). Psoriasis, Necrobiosis Lipoidica, Granuloma Annulare, Vitiligo and Skin Infections in the Same Diabetic Patient. J. Dermatol..

[50] Nakamura-Wakatsuki T., Yamamoto T. (2014). Palmoplantar Pustulosis Associated with Necrobiosis Lipoidica: A Possible Role of Tumor Necrosis Factor-α and Interleukin-17. J. Dermatol..

[51] Sibbald C., Reid S., Alavi A. (2015). Necrobiosis Lipoidica. Dermatol. Clin..

[52] Zhang S., Ke Z., Yang C., Zhou P., Jiang H., Chen L., Li Y., Li Q. (2021). High Glucose Causes Distinct Expression Patterns of Primary Human Skin Cells by RNA Sequencing. Front. Endocrinol..

[53] Oikarinen A., Mörtenhumer M., Kallioinen M., Savolainen E.R. (1987). Necrobiosis Lipoidica: Ultrastructural and Biochemical Demonstration of a Collagen Defect. J. Investig. Dermatol..

[54] Holland C., Givens V., Smoller B.R. (2001). Expression of the Human Erythrocyte Glucose Transporter Glut-1 in Areas of Sclerotic Collagen in Necrobiosis Lipoidica. J. Cutan. Pathol..

[55] Manicone A.M., McGuire J.K. (2008). Matrix Metalloproteinases as Modulators of Inflammation. Semin. Cell Dev. Biol..

[56] Jang D.-I., Lee A.-H., Shin H.-Y., Song H.-R., Park J.-H., Kang T.-B., Lee S.-R., Yang S.-H. (2021). The Role of Tumor Necrosis Factor Alpha (TNF-α) in Autoimmune Disease and Current TNF-α Inhibitors in Therapeutics. Int. J. Mol. Sci..

[57] Martinez F.O. (2011). Regulators of Macrophage Activation. Eur. J. Immunol..

[58] Wakusawa C., Fujimura T., Kambayashi Y., Furudate S., Hashimoto A., Aiba S. (2013). Pigmented Necrobiosis Lipoidica Accompanied by Insulin-Dependent Diabetes Mellitus Induces CD163^+^ Proinflammatory Macrophages and Interleukin-17-Producing Cells. Acta Derm. Venereol..

[59] Hassoun L.A., Sivamani R.K., Sharon V.R., Silverstein M.A., Burrall B.A., Tartar D.M. (2017). Ustekinumab to Target Granulomatous Dermatitis in Recalcitrant Ulcerative Necrobiosis Lipoidica: Case Report and Proposed Mechanism. Dermatol. Online J..

[60] Pourang A., Sivamani R.K. (2019). Treatment-Resistant Ulcerative Necrobiosis Lipoidica in a Diabetic Patient Responsive to Ustekinumab. Dermatol. Online J..

[61] Gibson R.S., Salian P., Beckles A., Stavert R., Tahan S., Kimball A.B., Porter M.L. (2023). Treatment of Necrobiosis Lipoidica with Secukinumab (Cosentyx): A Case Series. Int. J. Dermatol..

[62] Beatty P., Killion L., Blake C., Kelly G., Tobin A. (2021). Ulcerating Necrobiosis Lipoidica Successfully Treated with Ustekinumab. Australas. J. Dermatol..

[63] McPhie M.L., Swales W.C., Gooderham M.J. (2021). Improvement of Granulomatous Skin Conditions with Tofacitinib in Three Patients: A Case Report. SAGE Open Med. Case Rep..

[64] Oikonomou E.K., Antoniades C. (2019). The Role of Adipose Tissue in Cardiovascular Health and Disease. Nat. Rev. Cardiol..

[65] Taylor E.B. (2021). The Complex Role of Adipokines in Obesity, Inflammation, and Autoimmunity. Clin. Sci..

[66] Friedman J.M., Halaas J.L. (1998). Leptin and the Regulation of Body Weight in Mammals. Nature.

[67] La Cava A., Matarese G. (2004). The Weight of Leptin in Immunity. Nat. Rev. Immunol..

[68] Lord G.M., Matarese G., Howard J.K., Baker R.J., Bloom S.R., Lechler R.I. (1998). Leptin Modulates the T-Cell Immune Response and Reverses Starvation-Induced Immunosuppression. Nature.

[69] Matarese G., Moschos S., Mantzoros C.S. (2005). Leptin in Immunology. J. Immunol..

[70] De Rosa V., Procaccini C., Calì G., Pirozzi G., Fontana S., Zappacosta S., La Cava A., Matarese G. (2007). A Key Role of Leptin in the Control of Regulatory T Cell Proliferation. Immunity.

[71] Banks W.A. (2012). Role of the Blood–Brain Barrier in the Evolution of Feeding and Cognition. Ann. N. Y. Acad. Sci..

[72] Vasandani C., Clark G.O., Adams-Huet B., Quittner C., Garg A. (2017). Efficacy and Safety of Metreleptin Therapy in Patients with Type 1 Diabetes: A Pilot Study. Diabetes Care.

[73] Steppan C.M., Lazar M.A. (2002). Resistin and Obesity-Associated Insulin Resistance. Trends Endocrinol. Metab. TEM.

[74] Ikeda Y., Tsuchiya H., Hama S., Kajimoto K., Kogure K. (2013). Resistin Affects Lipid Metabolism during Adipocyte Maturation of 3T3-L1 Cells. FEBS J..

[75] Codoñer-Franch P., Alonso-Iglesias E. (2015). Resistin: Insulin Resistance to Malignancy. Clin. Chim. Acta Int. J..

[76] Filková M., Haluzík M., Gay S., Šenolt L. (2009). The Role of Resistin as a Regulator of Inflammation: Implications for Various Human Pathologies. Clin. Immunol..

[77] Kawashima K., Torii K., Furuhashi T., Saito C., Nishio E., Nishida E., Shintani Y., Morita A. (2011). Phototherapy Reduces Serum Resistin Levels in Psoriasis Patients. Photodermatol. Photoimmunol. Photomed..

[78] Boehncke S., Fichtlscherer S., Salgo R., Garbaraviciene J., Beschmann H., Diehl S., Hardt K., Thaçi D., Boehncke W.-H. (2011). Systemic Therapy of Plaque-Type Psoriasis Ameliorates Endothelial Cell Function: Results of a Prospective Longitudinal Pilot Trial. Arch. Dermatol. Res..

[79] Huang H., Shen E., Tang S., Tan X., Guo X., Wang Q., Ding H. (2015). Increased Serum Resistin Levels Correlate with Psoriasis: A Meta-Analysis. Lipids Health Dis..

[80] Geyikli I., Keskin M., Kör Y., Akan M. (2013). Increased Resistin Serum Concentrations in Patientswith Type 1 Diabetes Mellitus. J. Clin. Res. Pediatr. Endocrinol..

[81] Askin L., Abus S., Tanriverdi O. (2022). Resistin and Cardiovascular Disease: A Review of the Current Literature Regarding Clinical and Pathological Relationships. Curr. Cardiol. Rev..

[82] Briffa J.F., McAinch A.J., Poronnik P., Hryciw D.H. (2013). Adipokines as a Link between Obesity and Chronic Kidney Disease. Am. J. Physiol. Renal Physiol..

[83] Straub L.G., Scherer P.E. (2019). Metabolic Messengers: Adiponectin. Nat. Metab..

[84] Bai F., Zheng W., Dong Y., Wang J., Garstka M.A., Li R., An J., Ma H. (2017). Serum Levels of Adipokines and Cytokines in Psoriasis Patients: A Systematic Review and Meta-Analysis. Oncotarget.

[85] Gerdes S., Osadtschy S., Rostami-Yazdi M., Buhles N., Weichenthal M., Mrowietz U. (2012). Leptin, Adiponectin, Visfatin and Retinol-Binding Protein-4—Mediators of Comorbidities in Patients with Psoriasis?. Exp. Dermatol..

[86] Shibata S., Saeki H., Tada Y., Karakawa M., Komine M., Tamaki K. (2009). Serum High Molecular Weight Adiponectin Levels Are Decreased in Psoriasis Patients. J. Dermatol. Sci..

[87] Zhu K.-J., Shi G., Zhang C., Li M., Zhu C.-Y., Fan Y.-M. (2013). Adiponectin Levels in Patients with Psoriasis: A Meta-Analysis. J. Dermatol..

[88] Bavoso N.C., Pinto J.M., Soares M.M.S., Diniz M.d.S., Teixeira Júnior A.L. (2019). Psoriasis in Obesity: Comparison of Serum Levels of Leptin and Adiponectin in Obese Subjects—Cases and Controls. An. Bras. Dermatol..

[89] Baran A., Flisiak I., Jaroszewicz J., Świderska M. (2015). Effect of Psoriasis Activity on Serum Adiponectin and Leptin Levels. Adv. Dermatol. Allergol. Dermatol. Alergol..

[90] Chan W.S.A., Liew C.F., Theng C.T.S., Oon H.H. (2020). Serum Adiponectin Levels and Their Association with Cardiometabolic Risk Factors in Patients with Psoriasis. Cureus.

[91] Pereira R.I., Snell-Bergeon J.K., Erickson C., Schauer I.E., Bergman B.C., Rewers M., Maahs D.M. (2012). Adiponectin Dysregulation and Insulin Resistance in Type 1 Diabetes. J. Clin. Endocrinol. Metab..

[92] Begum M., Choubey M., Tirumalasetty M.B., Arbee S., Mohib M.M., Wahiduzzaman M., Mamun M.A., Uddin M.B., Mohiuddin M.S. (2023). Adiponectin: A Promising Target for the Treatment of Diabetes and Its Complications. Life.

[93] Ghayur T., Banerjee S., Hugunin M., Butler D., Herzog L., Carter A., Quintal L., Sekut L., Talanian R., Paskind M. (1997). Caspase-1 Processes IFN-Gamma-Inducing Factor and Regulates LPS-Induced IFN-Gamma Production. Nature.

[94] Sugawara S., Uehara A., Nochi T., Yamaguchi T., Ueda H., Sugiyama A., Hanzawa K., Kumagai K., Okamura H., Takada H. (2001). Neutrophil Proteinase 3-Mediated Induction of Bioactive IL-18 Secretion by Human Oral Epithelial Cells. J. Immunol..

[95] Román-Domínguez L., Salazar-León J., Meza-Sosa K.F., Pérez-Martínez L., Pedraza-Alva G. (2024). Adipose Tissue IL-18 Production Is Independent of Caspase-1 and Caspase-11. Immun. Inflamm. Dis..

[96] Rex D., Agarwal N., Prasad T.S.K., Kandasamy R.K., Subbannayya Y., Pinto S.M. (2020). A Comprehensive Pathway Map of IL-18-Mediated Signalling. J. Cell Commun. Signal..

[97] Esmailbeig M., Ghaderi A. (2017). Interleukin-18: A Regulator of Cancer and Autoimmune Diseases. Eur. Cytokine Netw..

[98] Harrison O.J., Srinivasan N., Pott J., Schiering C., Krausgruber T., Ilott N.E., Maloy K.J. (2015). Epithelial-Derived IL-18 Regulates Th17 Cell Differentiation and Foxp3^+^ Treg Cell Function in the Intestine. Mucosal Immunol..

[99] Ohta Y., Hamada Y., Katsuoka K. (2001). Expression of IL-18 in Psoriasis. Arch. Dermatol. Res..

[100] Companjen A., van der Wel L., van der Fits L., Laman J., Prens E. (2004). Elevated Interleukin-18 Protein Expression in Early Active and Progressive Plaque-Type Psoriatic Lesions. Eur. Cytokine Netw..

[101] Gangemi S., Merendino R.A., Guarneri F., Minciullo P.L., DiLorenzo G., Pacor M., Cannavò S.P. (2003). Serum Levels of Interleukin-18 and s-ICAM-1 in Patients Affected by Psoriasis: Preliminary Considerations. J. Eur. Acad. Dermatol. Venereol. JEADV.

[102] Arican O., Aral M., Sasmaz S., Ciragil P. (2005). Serum Levels of TNF-Alpha, IFN-Gamma, IL-6, IL-8, IL-12, IL-17, and IL-18 in Patients with Active Psoriasis and Correlation with Disease Severity. Mediat. Inflamm..

[103] Flisiak I., Klepacki A., Chodynicka B. (2006). Plasma and Scales Levels of Interleukin 18 in Comparison with Other Possible Clinical and Laboratory Biomarkers of Psoriasis Activity. Biomarkers.

[104] Marleau A.M., Sarvetnick N.E. (2011). IL-18 Is Required for Self-Reactive T Cell Expansion in NOD Mice. J. Autoimmun..

[105] Kretowski A., Mironczuk K., Karpinska A., Bojaryn U., Kinalski M., Puchalski Z., Kinalska I. (2002). Interleukin-18 Promoter Polymorphisms in Type 1 Diabetes. Diabetes.

[106] Harms R.Z., Yarde D.N., Guinn Z., Lorenzo-Arteaga K.M., Corley K.P., Cabrera M.S., Sarvetnick N.E. (2015). Increased Expression of IL-18 in the Serum and Islets of Type 1 Diabetics. Mol. Immunol..

[107] Vandanmagsar B., Youm Y.-H., Ravussin A., Galgani J.E., Stadler K., Mynatt R.L., Ravussin E., Stephens J.M., Dixit V.D. (2011). The NLRP3 Inflammasome Instigates Obesity-Induced Inflammation and Insulin Resistance. Nat. Med..

[108] Teupe B., Bergis K. (1991). Epidemiological Evidence for “Double Diabetes”. Lancet.

[109] Merger S.R., Kerner W., Stadler M., Zeyfang A., Jehle P., Müller-Korbsch M., Holl R.W., DPV Initiative, German BMBF Competence Network Diabetes Mellitus (2016). Prevalence and Comorbidities of Double Diabetes. Diabetes Res. Clin. Pract..

[110] Lee A.S., Twigg S.M., Flack J.R. (2021). Metabolic Syndrome in Type 1 Diabetes and Its Association with Diabetes Complications. Diabet. Med. J. Br. Diabet. Assoc..

[111] Mørk F.B., Madsen J.O.B., Pilgaard K.A., Jensen A.K., Klakk H., Tarp J., Bugge A., Heidemann M., Van Hall G., Pociot F. (2022). The Metabolic Syndrome Is Frequent in Children and Adolescents with Type 1 Diabetes Compared to Healthy Controls. Pediatr. Diabetes.

[112] Castro-Correia C., Santos-Silva R., Pinheiro M., Costa C., Fontoura M. (2018). Metabolic Risk Factors in Adolescent Girls with Type 1 Diabetes. J. Pediatr. Endocrinol. Metab. JPEM.

[113] Wolosowicz M., Lukaszuk B., Chabowski A. (2020). The Causes of Insulin Resistance in Type 1 Diabetes Mellitus: Is There a Place for Quaternary Prevention?. Int. J. Environ. Res. Public Health.

[114] Kim J., Oh C.-M., Kim H. (2023). The Interplay of Adipokines and Pancreatic Beta Cells in Metabolic Regulation and Diabetes. Biomedicines.

[115] Cnop M., Havel P.J., Utzschneider K.M., Carr D.B., Sinha M.K., Boyko E.J., Retzlaff B.M., Knopp R.H., Brunzell J.D., Kahn S.E. (2003). Relationship of Adiponectin to Body Fat Distribution, Insulin Sensitivity and Plasma Lipoproteins: Evidence for Independent Roles of Age and Sex. Diabetologia.

[116] Wang Y., Li Y., Qiao J., Li N., Qiao S. (2019). AMPK A1 Mediates the Protective Effect of Adiponectin against Insulin Resistance in INS-1 Pancreatic β Cells. Cell Biochem. Funct..

[117] Gao C., Zhao D., Qiu J., Zhang C., Ji C., Chen X., Liu F., Guo X. (2009). Resistin Induces Rat Insulinoma Cell RINm5F Apoptosis. Mol. Biol. Rep..

[118] Chetboun M., Abitbol G., Rozenberg K., Rozenfeld H., Deutsch A., Sampson S.R., Rosenzweig T. (2012). Maintenance of Redox State and Pancreatic Beta-Cell Function: Role of Leptin and Adiponectin. J. Cell. Biochem..

[119] Mohallem R., Aryal U.K. (2020). Regulators of TNFα Mediated Insulin Resistance Elucidated by Quantitative Proteomics. Sci. Rep..

[120] Gisondi P., Del Giglio M., Di Francesco V., Zamboni M., Girolomoni G. (2008). Weight Loss Improves the Response of Obese Patients with Moderate-to-Severe Chronic Plaque Psoriasis to Low-Dose Cyclosporine Therapy: A Randomized, Controlled, Investigator-Blinded Clinical Trial. Am. J. Clin. Nutr..

[121] Zhu K.-J., Zhang C., Li M., Zhu C.-Y., Shi G., Fan Y.-M. (2013). Leptin Levels in Patients with Psoriasis: A Meta-Analysis. Clin. Exp. Dermatol..

[122] Nestle F.O., Kaplan D.H., Barker J. (2009). Psoriasis. N. Engl. J. Med..

[123] Girolomoni G., Strohal R., Puig L., Bachelez H., Barker J., Boehncke W., Prinz J. (2017). The Role of IL-23 and the IL-23/T17 Immune Axis in the Pathogenesis and Treatment of Psoriasis. J. Eur. Acad. Dermatol. Venereol..

[124] Luan M., Shang Z., Teng Y., Chen X., Zhang M., Lv H., Zhang R. (2017). The Shared and Specific Mechanism of Four Autoimmune Diseases. Oncotarget.

[125] Shoelson S.E., Lee J., Goldfine A.B. (2006). Inflammation and Insulin Resistance. J. Clin. Investig..

[126] Armstrong A.W., Harskamp C.T., Armstrong E.J. (2013). Psoriasis and Metabolic Syndrome: A Systematic Review and Meta-Analysis of Observational Studies. J. Am. Acad. Dermatol..

[127] Mizutani H., Fukushima S., Masuguchi S., Yamashita J., Miyashita A., Nakahara S., Aoi J., Inoue Y., Jinnin M., Ihn H. (2013). Serum Levels of Leptin Receptor in Patients with Malignant Melanoma as a New Tumor Marker. Exp. Dermatol..

[128] Hwang J., Yoo J.A., Yoon H., Han T., Yoon J., An S., Cho J.Y., Lee J. (2021). The Role of Leptin in the Association between Obesity and Psoriasis. Biomol. Ther..

[129] Dopytalska K., Baranowska-Bik A., Roszkiewicz M., Bik W., Walecka I. (2020). The Role of Leptin in Selected Skin Diseases. Lipids Health Dis..

[130] Chen Y.-J., Wu C.-Y., Shen J.-L., Chu S.-Y., Chen C.-K., Chang Y.-T., Chen C.-M. (2008). Psoriasis Independently Associated with Hyperleptinemia Contributing to Metabolic Syndrome. Arch. Dermatol..

[131] Langan S.M., Seminara N.M., Shin D.B., Troxel A.B., Kimmel S.E., Mehta N.N., Margolis D.J., Gelfand J.M. (2012). Prevalence of Metabolic Syndrome in Patients with Psoriasis: A Population-Based Study in the United Kingdom. J. Investig. Dermatol..

[132] Gyldenløve M., Storgaard H., Holst J.J., Vilsbøll T., Knop F.K., Skov L. (2015). Patients with Psoriasis Are Insulin Resistant. J. Am. Acad. Dermatol..

[133] Libby P. (2002). Inflammation in Atherosclerosis. Nature.

[134] Vata D., Tarcau B.M., Popescu I.A., Halip I.A., Patrascu A.I., Gheuca Solovastru D.-F., Mocanu M., Chiriac P.C., Gheuca Solovastru L. (2023). Update on Obesity in Psoriasis Patients. Life.

[135] Mehta N.N., Yu Y., Pinnelas R., Krishnamoorthy P., Shin D.B., Troxel A.B., Gelfand J.M. (2011). Attributable Risk Estimate of Severe Psoriasis on Major Cardiovascular Events. Am. J. Med..

[136] Gisondi P., Tessari G., Conti A., Piaserico S., Schianchi S., Peserico A., Giannetti A., Girolomoni G. (2007). Prevalence of Metabolic Syndrome in Patients with Psoriasis: A Hospital-based Case–Control Study. Br. J. Dermatol..

[137] Naldi L., Addis A., Chimenti S., Giannetti A., Picardo M., Tomino C., Maccarone M., Chatenoud L., Bertuccio P., Caggese E. (2008). Impact of Body Mass Index and Obesity on Clinical Response to Systemic Treatment for Psoriasis. Evidence from the Psocare Project. Dermatology.

[138] Herron M.D., Hinckley M., Hoffman M.S., Papenfuss J., Hansen C.B., Callis K.P., Krueger G.G. (2005). Impact of Obesity and Smoking on Psoriasis Presentation and Management. Arch. Dermatol..

[139] Setty A.R., Curhan G., Choi H.K. (2007). Obesity, Waist Circumference, Weight Change, and the Risk of Psoriasis in Women: Nurses’ Health Study II. Arch. Intern. Med..

[140] Kirichenko T.V., Markina Y.V., Bogatyreva A.I., Tolstik T.V., Varaeva Y.R., Starodubova A.V. (2022). The Role of Adipokines in Inflammatory Mechanisms of Obesity. Int. J. Mol. Sci..

[141] Upala S., Sanguankeo A. (2015). Effect of Lifestyle Weight Loss Intervention on Disease Severity in Patients with Psoriasis: A Systematic Review and Meta-Analysis. Int. J. Obes..

[142] Codazzi V., Frontino G., Galimberti L., Giustina A., Petrelli A. (2024). Mechanisms and Risk Factors of Metabolic Syndrome in Children and Adolescents. Endocrine.

[143] Chait A., den Hartigh L.J. (2020). Adipose Tissue Distribution, Inflammation and Its Metabolic Consequences, Including Diabetes and Cardiovascular Disease. Front. Cardiovasc. Med..

[144] O’Callaghan V.S., Hansell N.K., Guo W., Carpenter J.S., Shou H., Strike L.T., Crouse J.J., McAloney K., McMahon K.L., Byrne E.M. (2021). Genetic and Environmental Influences on Sleep-Wake Behaviors in Adolescence. Sleep Adv. J. Sleep Res. Soc..

[145] Reisinger C., Nkeh-Chungag B.N., Fredriksen P.M., Goswami N. (2021). The Prevalence of Pediatric Metabolic Syndrome-a Critical Look on the Discrepancies between Definitions and Its Clinical Importance. Int. J. Obes..

[146] Gelfand J.M., Neimann A.L., Shin D.B., Wang X., Margolis D.J., Troxel A.B. (2006). Risk of Myocardial Infarction in Patients with Psoriasis. JAMA.

[147] Pietrzak A., Grywalska E., Walankiewicz M., Lotti T., Roliński J., Myśliński W., Chabros P., Piekarska-Myślińska D., Reich K. (2017). Psoriasis and Metabolic Syndrome in Children: Current Data. Clin. Exp. Dermatol..

[148] Lakshmi H.V.S., Budamakuntla L., Sundar C.M.S. (2023). Prevalence of Metabolic Syndrome among Children with Psoriasis in Urban Bengaluru. Clin. Dermatol. Rev..

[149] Goldminz A.M., Buzney C.D., Kim N., Au S.-C., Levine D.E., Wang A.C., Volf E.M., Yaniv S.S., Kerensky T.A., Bhandarkar M. (2013). Prevalence of the Metabolic Syndrome in Children with Psoriatic Disease. Pediatr. Dermatol..

[150] Hugh J., Voorhees A.S.V., Nijhawan R.I., Bagel J., Lebwohl M., Blauvelt A., Hsu S., Weinberg J.M. (2014). From the Medical Board of the National Psoriasis Foundation: The Risk of Cardiovascular Disease in Individuals with Psoriasis and the Potential Impact of Current Therapies. J. Am. Acad. Dermatol..

[151] Sinagra E., Perricone G., Romano C., Cottone M. (2013). Heart Failure and Anti Tumor Necrosis Factor-Alpha in Systemic Chronic Inflammatory Diseases. Eur. J. Intern. Med..

[152] Chang S.H., Dong C. (2009). IL-17F: Regulation, Signaling and Function in Inflammation. Cytokine.

[153] Reich K., Warren R.B., Lebwohl M., Gooderham M., Strober B., Langley R.G., Paul C., Cuyper D.D., Vanvoorden V., Madden C. (2021). Bimekizumab versus Secukinumab in Plaque Psoriasis. N. Engl. J. Med..

[154] Warren R.B., Lebwohl M., Thaçi D., Gooderham M., Pinter A., Paul C., Gisondi P., Szilagyi B., White K., Deherder D. (2025). Bimekizumab Efficacy and Safety through 3 Years in Patients with Moderate to Severe Plaque Psoriasis: Long-Term Results from the BE RADIANT Phase 3b Trial Open-Label Extension Period. Br. J. Dermatol..

[155] Morey A., Loades M.E. (2021). Review: How Has Cognitive Behaviour Therapy Been Adapted for Adolescents with Comorbid Depression and Chronic Illness? A Scoping Review. Child Adolesc. Ment. Health.

